# Vaccinia virus lacking the deoxyuridine triphosphatase gene (F2L) replicates well in vitro and in vivo, but is hypersensitive to the antiviral drug (N)-methanocarbathymidine

**DOI:** 10.1186/1743-422X-5-39

**Published:** 2008-03-05

**Authors:** Mark N Prichard, Earl R Kern, Debra C Quenelle, Kathy A Keith, Richard W Moyer, Peter C Turner

**Affiliations:** 1 Department of Pediatrics, University of Alabama School of Medicine, Birmingham, AL 35233, USA; 2 Department of Molecular Genetics and Microbiology, University of Florida College of Medicine, Gainesville, FL 32610, USA

## Abstract

**Background:**

The vaccinia virus (VV) F2L gene encodes a functional deoxyuridine triphosphatase (dUTPase) that catalyzes the conversion of dUTP to dUMP and is thought to minimize the incorporation of deoxyuridine residues into the viral genome. Previous studies with with a complex, multigene deletion in this virus suggested that the gene was not required for viral replication, but the impact of deleting this gene alone has not been determined in vitro or in vivo. Although the crystal structure for this enzyme has been determined, its potential as a target for antiviral therapy is unclear.

**Results:**

The F2L gene was replaced with GFP in the WR strain of VV to assess its effect on viral replication. The resulting virus replicated well in cell culture and its replication kinetics were almost indistinguishable from those of the wt virus and attained similar titers. The virus also appeared to be as pathogenic as the WR strain suggesting that it also replicated well in mice. Cells infected with the dUTPase mutant would be predicted to affect pyrimidine deoxynucleotide pools and might be expected to exhibit altered susceptibility to pyrimidine analogs. The antiviral activity of cidofovir and four thymidine analogs were evaluated both in the mutant and the parent strain of this virus. The dUTPase knockout remained fully susceptible to cidofovir and idoxuridine, but was hypersensitive to the drug (N)-methanocarbathymidine, suggesting that pyrimidine metabolism was altered in cells infected with the mutant virus. The absence of dUTPase should reduce cellular dUMP pools and may result in a reduced conversion to dTMP by thymidylate synthetase or an increased reliance on the salvage of thymidine by the viral thymidine kinase.

**Conclusion:**

We confirmed that F2L was not required for replication in cell culture and determined that it does not play a significant role on virulence of the virus in intranasally infected mice. The recombinant virus is hypersensitive to (N)-methanocarbathymidine and may reflect metabolic differences in the mutant virus.

## Background

All free-living organisms have mechanisms to minimize the incorporation of uracil in their genomes. These residues in DNA can arise either through misincorporation of dUTP by DNA polymerase or the spontaneous deamination of cytosine and can result in A:T transition mutations in one of the nascent strands [1]. Minimizing the incorporation of these bases and excising those that arise prevents the accumulation of deleterious mutations. The enzymes uracil DNA glycosylase (UNG) and deoxyuridine triphosphatase (dUTPase) arose very early in evolutionary terms and act in concert to protect organisms from uracil residues [2,3]. Enzymes with dUTPase activity catalyze the dephosphorylation of dUTP to minimize its incorporation into genomic DNA, while UNG family members repair uracil residues from DNA by base excision repair.

These protective enzymes are also present in many viruses including retroviruses, herpesviruses and orthopoxviruses [4]. Proteins with UNG activity are either encoded by these viruses, or recruited by viral proteins and are thought to be important in viral replication [1]. Similarly, dUTPase homologs are encoded by many lentiviruses, as well as all herpesviruses and orthopoxviruses and are presumed to minimize potential damage by the incorporation of uracil residues [3]. Both herpes simplex virus (HSV) and vaccinia virus (VV) encode homologs of dUTPase and the viral enzymes hydrolyze dUTP to dUMP and require divalent cations for their activity [5,6]. The dUTPase encoded by the F2L gene of VV is a 16.5 kiloDalton protein that forms homotrimers and enzymatic studies determined that the *K*_*m *_for dUTP was 1 μM and that it was competitively inhibited by 8-azido-ATP [7]. Recently, the crystal structure of this trimeric enzyme was determined and proved to be closely related to that of the human homolog, although the central channel was somewhat larger in the viral enzyme. These results suggested that the development of specific inhibitors of this enzyme might be possible [8].

If the dUTPase fulfills an essential role in viral replication then inhibitors of this enzyme might have the potential to be used in the treatment of orthopoxvirus infections. One previous report described a recombinant virus with a large deletion resulting in the elimination of 55 open reading frames including F2L [9]. This recombinant was viable suggesting that the dUTPase was not required for replication in cell culture, although it did not exclude the possibility that it might be important for replication in vivo. In HSV, deletion of the dUTPase homolog did not effect the replication of the virus in vitro, however its virulence was reduced by 1000-fold in mice following footpad inoculation and reduced replication in the CNS was also observed [10]. To assess the potential of the dUTPase as a target for antiviral therapy, F2L was deleted in the WR strain of VV and the replication of the mutant virus (VV Δ F2L-gfp) was evaluated in cell culture and in intranasally infected mice. The deletion of F2L had a minimal impact on viral replication in vitro and did not appear to significantly reduce the virulence in vivo suggesting that it did not contribute appreciably to disease, and that it was not a good target for the development of antiviral therapies.

## Results

**Figure 1:**
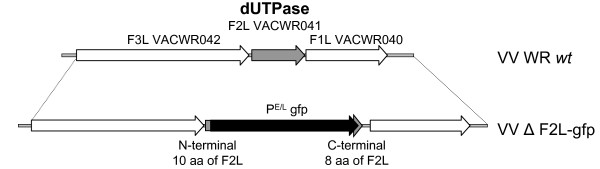
Genomic structure of the F2L region of VV Δ F2L-gfp. The F2L gene in VV strain WR (shaded arrow in the top line) was replaced with the gfp gene driven by the synthetic E/L promoter (black arrow in the bottom line). The resulting virus was designated VV Δ F2L-gfp and contained a deletion in F2L corresponding to amino acids 11–129 of the open reading frame.

**Figure 2:**
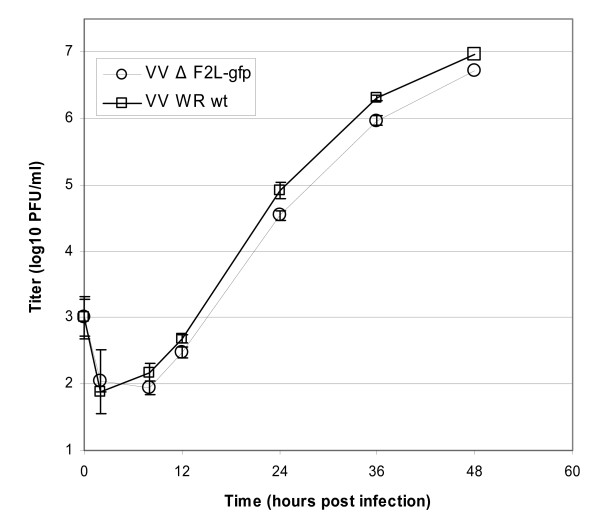
Replication kinetics of VV Δ F2L-gfp in HFF cells. Triplicate wells of 6-well plates were infected with the WR strain of VV (square symbols) or VV Δ F2L-gfp (circular symbols). Virus from each well was harvested at 2, 8, 12, 24, 36, and 48 h post infection. All samples including inocula were titered in duplicate and average titers are shown with error bars representing the standard deviations.

A recombinant virus lacking only the dUTPase gene was constructed by homologous recombination in the WR strain of VV. In this virus, F2L was replaced with the gfp gene driven by the synthetic E/L promoter. Fluorescent recombinant plaques were plaque purified three times to eliminate any contaminating wild type (*wt*) parental virus. The insertion of the gfp resulted in the deletion of most of the F2L open reading frame from amino acids 11–129 (Fig. 1). The engineered mutation did not appear to affect plaque size and confirmed that F2L was not required for replication in cell culture (data not shown). The replication kinetics of this virus were assessed in human foreskin fibroblast (HFF) cells at an MOI of 0.001 PFU/cell. Results from this experiment suggested that the F2L mutant (VVΔF2L-gfp) replicated well in these cells and yielded titers that were reproducibly reduced compared to those of the parent virus (Fig. 2). However, the slight impairment in replication was so minor that it is unlikely to be an important factor in cell culture. These results suggested that the dUTPase is not required for the replication of VV in cell culture and are consistent with the previous report.

Although deletion of the dUTPase gene did not appear to affect replication in cell culture, it is possible that it may play a significant role in vivo as has been seen with the dUTPase knockout virus in HSV. To test this hypothesis, 3 week old female, BALB/c mice were anesthetized with ketamine-xylazine and inoculated intranasally with 10-fold dilutions of the viruses in a volume of 40 μl (20 μl per nostril). Two isolates of wt VV WR, and VV Δ F2L-gfp were evaluated in this experiment to assess the virulence characteristic of these strains. Mice were observed daily for 21 days and were evaluated for clinical signs of infection and mortality. Infection with 1.6 × 10^4 ^PFU or greater resulted in 100% mortality, while some animals survived with 10-fold less virus (Table 1). Thus, no significant differences were observed in the virulence among the two isolates of WR and the mutant virus. These results suggested that dUTPase is not required for virulence in mice and its removal does not appear to impact viral replication in animals.

**Table 1:** VV Δ F2L-gfp exhibits virulence characteristics that are similar to the parent virus.

Virus (PFU/mouse)^a^	Mortality		MDD^b^
	Number	Percent	
**VV-WR, UAB**			
1.6 × 10^4^	10/10	100	6.8 ± 0.8
1.6 × 10^3^	3/10	30	8.3 ± 0.6
1.6 × 10^2^	0/10	0	---
16	0/10	0	---
1.6	0/10	0	---
			
**VV-WR, Turner**			
Stock 1.6 × 10^8^	10/10	100	3.2 ± 0.4
1.6 × 10^7^	10/10	100	3.9 ± 0.3
1.6 × 10^6^	10/10	100	5.1 ± 0.3
1.6 × 10^5^	10/10	100	6.2 ± 0.4
1.6 × 10^4^	10/10	100	7.9 ± 1.0
			
**VV Δ F2L-gfp**			
Stock 6 × 10^7^	10/10	100	4.2 ± 0.4
6 × 10^6^	10/10	100	4.7 ± 0.5
6 × 10^5^	10/10	100	6.1 ± 0.6
6 × 10^4^	10/10	100	7.1 ± 0.3
6 × 10^3^	5/10	50	8.4 ± 0.5

a. Anesthetized mice were inoculated intranasally with 40 μl of virus (20 μl/nostril).

b. Mean day of death (MDD) is shown with the standard deviation.

Deletion of the dUTPase is predicted to effect pyrimidine metabolism in infected cells and may alter the susceptibility of the mutant virus to some antiviral drugs. A set of thymidine analogs was selected and a standard plaque reduction assay was used to evaluate the susceptibility of the dUTPase mutant and the parent virus. The mutant remained fully sensitive to all of the drugs tested including cidofovir (CDV), idoxuridine (IDU), and two thymidine analogs reported to require phosphorylation by the VV thymidine kinase (TK) [11]. The only significant difference observed in the mutant virus was the modest but repeatable increase in the efficacy of N-methanocarbathymidine (N-MCT) (Table 2). This compound is a carbocyclic thymidine analog that inhibits the replication of VV both in vitro and in vivo [12,13], and also appears to require phosphorylation by the viral TK [12].

**Table 2:** Susceptibility of VV Δ F2L-gfp to selected thymidine analogs and cidofovir.

Compound Name	VV Δ F2L-gfp (EC_50_, μM)^a^	VV WR *wt *(EC_50_, μM)
IDU	2.5 ± 0.9	2.4 ± 0.6
N-MCT	6.2 ± 3.5	12 ± 1.6
PFT3	2.0 ± 0.6	2.4 ± 0.4
PFT4	2.3 ± 1.1	2.5 ± 0.1
CDV	11 ± 4.2	10 ± 0.3

a. Concentration required to reduce plaque formation by 50%. Values shown are the average of duplicate determinations with the standard deviations shown.

## Discussion

Results presented here are consistent with a previous report that showed that the dUTPase was not required for replication in cell culture using a virus containing a multigene deletion [9]. They also suggest that the function of this protein is of modest importance in the replication of VV in vivo. These results contrast with those reported previously for the dUTPase negative mutants of HSV where virulence was severely affected in mice [10]. It is unclear if the observed differences in replication in mice reflect real differences in the biology of these viruses, or are related to differences in the animal models including route of infection. In HSV, direct intracranial inoculation resulted in a 10-fold reduction in virulence of the mutant, whereas footpad inoculation increased the LD_50 _from approximately 10^3 ^PFU to more than 10^6 ^PFU of the mutant virus. Thus, the observed virulence of the HSV mutant was dependent on the route of administration and it is unclear if reduced virulence would be observed following intranasal inoculation.

We show here that there is little if any attenuation when mice are intranasally infected with VV in which the dUTPase has been deleted. Additional experiments are required to resolve this issue and the rabbit model of VV infection and might be a more sensitive indicator of reduced virulence associated with the mutant virus [14]. It is also possible that reduced virulence might be observed if infection was initiated through inoculation at peripheral sites.

**Figure 3:**
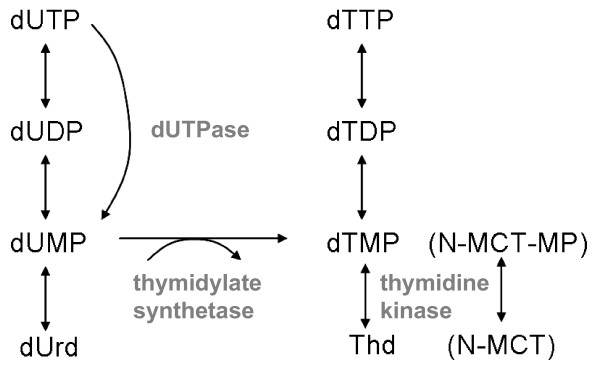
Model of dUTPase function in VV and a potential explanation of (N)-MCT hypersensitivity. Deletion of dUTPase is predicted to reduce intracellular pools of its dUMP product, which is converted to dTMP by thymidylate synthetase. This is significant since dTMP is predicted to compete with the monophosphate metabolite of N-MCT (N-MCT-MP) for subsequent anabolic reactions or as substrates for the target enzyme.

Differences in pyrimidine metabolism were predicted to occur in the absence of the dUTPase so a set of selected thymidine analogs were used as potential indicators of metabolic differences. The efficacy of the CDV control virus was unchanged in the mutant, as was the activity of IDU and two thymidine analogs reported previously [11]. However, the mutation in VV ΔF2L-gfp appeared to confer some hypersensitivity to the drug (N)-MCT. The mechanism of action of this compound is incompletely understood, although it appears to require phosphorylation by the viral TK to the monophosphate (N-MCT-MP) [12]. This is significant since intracellular pools of dUMP and dTMP are predicted to be reduced in the absence of the viral dUTPase. Thus, the increased ratios of N-MCT-MP:dTMP and N-MCT-MP:dUMP should reduce competition for subsequent anabolic reactions or as substrates for the target enzyme, perhaps thymidylate synthetase (Fig. 3). It is unclear why this was not observed with IDU and the other compounds, but it likely reflects the specific mechanisms of the individual drugs.

## Conclusion

Data presented here suggest that VV dUTPase is not required for viral replication in cell culture. The deletion of F2L also does not appear to impact the virulence of the virus in mice following intranasal infection. These studies suggest that dUTPase is not a particularly good target for the development of antiviral therapies, although it remains possible that other animal models may identify an important function of this enzyme. The mutation does not affect the efficacy of most antiviral drugs including CDV although it appears to confer a modest hypersensitivity to N-MCT and may reflect metabolic differences in the mutant virus.

## Methods

### Cells, viruses, and drugs

Recombinant virus designated VV ΔF2L-gfp and the parental *wt *VV strain WR (Turner) were received from Dr. Pete Turner, University of Florida, Gainesville, FL. VV strain WR (UAB) used in the animal studies was obtained from the American Type Culture Collection (ATCC), Manassas, VA. Working stocks of both VV-WR isolates and VV ΔF2L-gfp were propagated in Vero cells obtained from ATCC. Human foreskin fibroblasts were prepared as primary cultures from freshly obtained newborn human foreskins as soon as possible after circumcision. Culture medium for both cell lines was minimum essential medium (MEM) with Earle's salts containing 10% fetal bovine serum and standard concentrations of L-glutamine, penicillin and gentamicin.

The drugs tested included CDV, IDU, N-MCT, 5-(2-amino-3-cyano-5-oxo-5,6,7,8-tetrahydro-4H-chromen-4-yl)-1-(2-deoxypento-furanosyl)-pyrimidine-2,4(1H,3H)-dione (PFT3), and 1-(2-deoxypentofuranosyl)-5- [(3-methyl-5-oxo-1-phenyl-4,5-dihydro-4H-pyrazol-4-ylidene)pyrimidine-2,4(1H,3H)-dione (PFT4). Compounds designated PFT3 and PFT4 were synthesized by Paul Torrence (Northern Arizona University) and were described previously [11]; N-MCT was described previously [15]; CDV was a gift of Mick Hitchcock at Gilead sciences and IDU was purchased from Sigma Aldrich (St. Louis, MO).

### Construction of VV ΔF2L-gfp

A recombinant was constructed from VV strain WR with most of F2L replaced with the gfp gene driven by the synthetic E/L promoter by methods similar to those described previously [16]. Primers IDT 715 (5'-ATGCTGCTTGGGTTAATATGCCG-3') against the F3L gene upstream from F2L and IDT 716 (GCGAAGCTTAACTGGTGAGTTAATATTCATGTTGAAC, *Hin*dIII site underlined) against the complement of F2L residues 3–31 were used to PCR amplify a 487-bp fragment upstream from F2L. A 531-bp PCR product consisting of the downstream flank from F2L was made using primers IDT 717 (GCGCTCGAGAGGGTTTGGATCAACAGGAC, *Xho*I site underlined) against F2L residues 417–436 and IDT 718 (CATACATCGTCTACCCAATTCGG) against F1L. *Hin*dIII-digested, dephosphorylated IDT 715+716 PCR product and *Xho*I-digested, dephosphorylated IDT 717+718 PCR product were ligated to a *Hin*dIII-*Xho*I restriction fragment consisting of the P^E/L ^promoter linked to the gfp gene. The ligation mix was PCR amplified with primers IDT 715 and IDT 718 to generate a 1.8-kb product of gfp flanked by portions of F3L and F1L. CV-1 cells were infected with wt VV-WR, transfected with the F3L-gfp-F1L DNA, and plaques expressing gfp were isolated by fluorescence. The resulting virus was designated VV ΔF2L-gfp and contained a deletion in F2L corresponding to amino acids 11–129 of the open reading frame. The genomic structure and purity of this virus was confirmed by PCR using primers IDT 715 + 718. No fragment of 1.4 kb corresponding to wt F2L plus flanks was detected, but a 1.8 kb fragment of F3L-gfp-F1L was present.

### Growth curves

To determine the in vitro replication of the viruses, HFF cells were incubated in 6 well plates for 24 h prior to infection at 37°C with 5% CO_2 _and 90% humidity. Triplicate wells were infected with *wt *VV-WR or VV Δ F2L-gfp at an MOI of 0.001. Infected plates were frozen at -80°C at 2, 8, 12, 24, 36 and 48 h post infection. Duplicate titrations of each of the triplicate wells were conducted in HFF cells in 6 well plates. Plaques were enumerated and titers were determined for each time point and virus.

### Determination of antiviral activity

Plaque reduction assays were performed using HFF cells added to six well plates and incubated 48 h prior to infection. On the day of assay, drug at two times the final desired concentration was diluted serially 1:5 in 2X MEM with 10% FBS to provide six concentrations. Aspiration of culture medium from triplicate wells for each drug concentration was followed by addition of 0.2 ml/well of diluted virus which would give 20–30 plaques per well in MEM containing 10% FBS or 0.2 ml medium for drug toxicity wells. The plates were incubated for one h with shaking every 15 minutes. An equal amount of 1% agarose was added to an equal volume of each drug dilution and this mixture was added to each well in 2 ml volumes and the plates incubated for three days. The cells were stained with a 0.02% solution of neutral red (Sigma, St. Louis, MO) in PBS and incubated for 5–6 h. The stain was aspirated, and plaques counted using a stereomicroscope at 10× magnification and 50% effective concentration (EC_50_) values were calculated by standard methods.

### Virulence

Three week old female, BALB/c mice were anesthetized with ketamine-xylazine and inoculated intranasally with 10-fold dilutions of the viruses in a volume of 40 μl (20 μl per nostril). Two isolates of WR, and VV Δ F2L-gfp were evaluated in this experiment to assess the virulence characteristic of these strains. Mice were observed daily for 21 days and were evaluated for clinical signs of infection and mortality.

## Abbreviations

Hour (h), wild type (wt), plaque forming unit (PFU), vaccinia virus (VV), herpes simplex virus (HSV), (N)-methanocarbathymidine (N-MCT), cidofovir (CDV), idoxuridine (IDU), N-MCT monophosphate (N-MCT-MP), deoxyuridine triphosphatase (dUTPase), deoxyuridine triphosphate (dUTP), deoxyuridine diphosphate (dUDP), deoxyuridine monophosphate (dUMP), green fluorescent protein (GFP), deoxythymidine monophosphated (TMP), uracil DNA glycosylase (UNG), human foreskin fibroblast (HFF), lethal dose 50% (LD_50_), effective concentration (EC_50_), American type culture collection (ATCC), 5-(2-amino-3-cyano-5-oxo-5,6,7,8-tetrahydro-4H-chromen-4-yl)-1-(2-deoxypento-furanosyl)-pyrimidine-2,4(1H,3H)-dione (PFT3), 1-(2-deoxypentofuranosyl)-5-[(3-methyl-5-oxo-1-phenyl-4,5-dihydro-4H-pyrazol-4-ylidene)pyrimidine-2,4(1H,3H)-dione (PFT4), minimum essential medium (MEM), central nervous system (CNS).

## Competing interests

The author(s) declare that they have no competing interests.

## Authors' contributions

MNP contributed to the design of experiments, analysis of the data and the drafted the manuscript. ERK contributed to the conception of the studies and the critical review of the manuscript. DCQ contributed to the design of the experiments and analysis of the data. KAK contributed to the acquisition and interpretation of data. RWM contributed to the conception of the studies and the critical review of the manuscript. PCT contributed to the design of experiments, the acquisition and analysis of data and the critical review of the manuscript.
